# Public Awareness of and Attitudes Toward the Use of AI in Pathology Research and Practice: Mixed Methods Study

**DOI:** 10.2196/59591

**Published:** 2025-04-02

**Authors:** Claire Lewis, Jenny Groarke, Lisa Graham-Wisener, Jacqueline James

**Affiliations:** 1 School of Medicine Dentistry and Biomedical Sciences Queen's University Belfast Belfast United Kingdom; 2 School of Psychology University of Galway Galway Ireland; 3 School of Psychology Queen's University Belfast Belfast United Kingdom

**Keywords:** artificial intelligence, AI, public opinion, pathology, health care, public awareness, survey

## Abstract

**Background:**

The last decade has witnessed major advances in the development of artificial intelligence (AI) technologies for use in health care. One of the most promising areas of research that has potential clinical utility is the use of AI in pathology to aid cancer diagnosis and management. While the value of using AI to improve the efficiency and accuracy of diagnosis cannot be underestimated, there are challenges in the development and implementation of such technologies. Notably, questions remain about public support for the use of AI to assist in pathological diagnosis and for the use of health care data, including data obtained from tissue samples, to train algorithms.

**Objective:**

This study aimed to investigate public awareness of and attitudes toward AI in pathology research and practice.

**Methods:**

A nationally representative, cross-sectional, web-based mixed methods survey (N=1518) was conducted to assess the UK public’s awareness of and views on the use of AI in pathology research and practice. Respondents were recruited via Prolific, an online research platform. To be eligible for the study, participants had to be aged >18 years, be UK residents, and have the capacity to express their own opinion. Respondents answered 30 closed-ended questions and 2 open-ended questions. Sociodemographic information and previous experience with cancer were collected. Descriptive and inferential statistics were used to analyze quantitative data; qualitative data were analyzed thematically.

**Results:**

Awareness was low, with only 23.19% (352/1518) of the respondents somewhat or moderately aware of AI being developed for use in pathology. Most did not support a diagnosis of cancer (908/1518, 59.82%) or a diagnosis based on biomarkers (694/1518, 45.72%) being made using AI only. However, most (1478/1518, 97.36%) supported diagnoses made by pathologists with AI assistance. The adjusted odds ratio (aOR) for supporting AI in cancer diagnosis and management was higher for men (aOR 1.34, 95% CI 1.02-1.75). Greater awareness (aOR 1.25, 95% CI 1.10-1.42), greater trust in data security and privacy protocols (aOR 1.04, 95% CI 1.01-1.07), and more positive beliefs (aOR 1.27, 95% CI 1.20-1.36) also increased support, whereas identifying more risks reduced the likelihood of support (aOR 0.80, 95% CI 0.73-0.89). In total, 3 main themes emerged from the qualitative data: *bringing the public along*, *the human in the loop*, and *more hard evidence needed*, indicating conditional support for AI in pathology with human decision-making oversight, robust measures for data handling and protection, and evidence for AI benefit and effectiveness.

**Conclusions:**

Awareness of AI’s potential use in pathology was low, but attitudes were positive, with high but conditional support. Challenges remain, particularly among women, regarding AI use in cancer diagnosis and management. Apprehension persists about the access to and use of health care data by private organizations.

## Introduction

### Background

The last decade has witnessed major advances in the development and application of artificial intelligence (AI) technologies. Coined by John McCarthy in 1955, AI was originally described as the “science and engineering of making intelligent machines, especially intelligent computer programs” [1]. It is already featured in many aspects of modern life, including the use of smart home devices, search engines, and facial recognition software. Indeed, given its potential to improve efficiency and productivity across a range of industries and public sectors, AI has been regarded as one of the most important new technologies of the century for policy makers, governments, and citizens alike [2].

In the context of health, it has been claimed that AI has the potential to revolutionize health care by offering improved diagnostic accuracy, reducing costs, and enabling a more personalized approach to medicine [3,4]. Currently, there are a limited number of AI applications that have been approved for clinical use in Europe and the United States, with most of these being in the field of medical imaging [5]. In 2021, the first AI-based pathology software was approved by the US Food and Drug Administration to assist with the detection of prostate cancer [6]. This milestone was indicative of the transformational shift that has occurred in pathology in the last few decades, a shift that has seen a move away from the traditional microscopic review of a glass slide toward the use of state-of-the-art digital scanners to generate a high-resolution image that is wholly representative of the original tissue section and of comparable diagnostic quality [7]. This digital approach offers many advantages in terms of improved efficiency and productivity in clinical laboratory workflows, but it is also the availability of complex visual information in these digitally generated images that has provided a rich source of data for the development of novel pathology AI algorithms to potentially aid with diagnostic and analytical procedures [8]. Many studies have since used these digitized slide images to train computer algorithms for aspects of pathology practice, including automated identification of lymph node metastases [9], prediction of prognosis [10], disease grading and classification [11,12], and identification of clinically actionable targets (biomarkers) for cancer treatment [13], with many studies claiming comparable or superior performance of AI to that of human intelligence [9-12].

The potential of these AI tools in terms of improved efficiency, cost-effectiveness, and accuracy of diagnosis cannot be underestimated. However, in addition to regulatory considerations, several challenges must be addressed in the development and implementation of such technologies in diagnostic pathology practice. One of these is public support for regulated access to health care datasets for AI research. The development of machine learning and deep learning approaches in pathology not only requires access to large numbers of digitized whole slide images (WSIs) but also requires the generation and aggregation of vast amounts of anonymized patient data, including but not limited to data from health care records and investigative tests. Therefore, the public must have trust and confidence that their data will be used safely, securely, and appropriately [14]. While safeguards are in place to govern the sharing of data [15], it is well documented that corporate profiteering and data privacy and security are consistently among the public’s top concerns regarding the sharing and use of health data in research [16-18]. High-profile cases such as the one involving the AI application Google DeepMind, which was granted “legally inappropriate” access to National Health Service (NHS) data, have tested public confidence in how the NHS safeguards access to data for secondary use [18]. Furthermore, evidence indicates that public support for the use of health data in AI research is conditional on the potential benefit in return [19]. This is important to note given that there appears to be a general lack of public knowledge and awareness of data science and AI [20], which could result in a failure to see the benefit and value of its application in a health care setting such as diagnostic pathology.

Second, while research in pathology algorithm development uses data in the form of digitized WSIs, the WSI must still be generated from a human tissue sample. In the United Kingdom, the use of human tissue samples for research is governed by the Human Tissue Act of 2004 [21]. This provides a regulatory framework that allows deidentified residual clinical material (eg, formalin-fixed paraffin-embedded tissue blocks residing in diagnostic archives and surplus to further clinical requirement) to be used for research purposes without explicit consent so long as the sample is rendered anonymous to the end user [21]. This position is in line with public consensus supporting the use of tissue samples in research [22-25] and particularly in cancer research [25]. However, we do know that various factors influence public attitudes toward the use of their samples in research. These include sociodemographics, health status, cultural and religious values, awareness, knowledge, past experiences, beliefs about the potential benefits and risks, attitudes toward genetic and medical research, experience with the health care system, and trust in government and scientific institutions [22-27]. These factors have yet to be explored in the context of the use of tissue and linked data in AI research.

### Objectives

Finally, there needs to be public support for the use of AI in their own health care, particularly in disciplines such as pathology, where it is predicted that AI will significantly transform practice [28]. One UK study surveyed men who had undergone a prostate biopsy (N=1276) and reported that respondents were mostly supportive of AI as a diagnostic companion to professional decision-making (1058/1276, 83%) [29]. However, more widely, there have been documented concerns from the public about an overreliance on AI in health care at the expense of human involvement [30,31] and uncertainty about the delegation of clinical responsibilities to machines and systems [32,33]. A report from the Academy of Medical Royal Colleges recommended early and ongoing public engagement in the development and implementation of AI to maximize impact and ensure that the public has their say when examining the acceptability of such technologies [34]. Currently, there is a dearth of evidence on which to draw such conclusions in pathology practice as there has been limited investigation of this to date beyond the male cohort surveyed by Rakovic et al [29]. To this end, there is a need to examine the extent of public awareness of AI and their views on its potential use in diagnostic pathology to gauge the level of support and identify any concerns, ultimately fostering informed public endorsement of the integration of AI applications in pathology and the anticipated benefits in terms of efficiency and accuracy. Therefore, the aim of this study was to investigate public awareness of and attitudes toward the use of AI in pathology research and practice with the following objectives: (1) to assess public awareness and attitudes regarding the *development* of AI technologies in pathology; (2) to assess public awareness and attitudes regarding the *implementation* of AI technologies in pathology; (3) to identify matters of concern and sources of support regarding the design and implementation of AI technologies in pathology; and (4) to determine whether sociodemographic characteristics, experiences with cancer, awareness, beliefs, and attitudes influence support for the development and implementation of AI in pathology.

## Methods

### Design

A nationally representative cross-sectional, web-based mixed methods survey was conducted to (1) assess the UK public’s awareness of and views on the use of AI in diagnostic pathology and (2) ascertain levels and predictors of support for the use of AI in diagnostic pathology and the use of deidentified personal health data and images of tissue in the development of AI technologies for cancer diagnosis and management.

### Recruitment

The survey was administered on the web (November 30, 2022), with respondents recruited via an online crowdsourcing platform managed by Prolific Academic Ltd [35] to provide a nationally representative sample of approximately 1500 UK respondents stratified across 3 demographics: age, gender, and ethnicity. To be eligible for this study, respondents had to reside in the United Kingdom, be aged >18 years, and have the capacity to express their own opinion.

### Survey Design and Measures

The survey was created and hosted on Qualtrics (Qualtrics International Inc), a web-based software to assist with survey management. The web-based survey consisted of 30 closed questions, 2 open questions, 2 attention checks, 2 screener validation questions (age and country of residence), and 1 CAPTCHA challenge (Multimedia Appendix 1). The survey was developed by the research team with input from a patient and public involvement panel and pilot-tested with individuals external to the research team (n=5). To ensure that the questions were easy to understand, lay terms such as *cancer diagnosis and management* were used in place of medical terminology. In the first section of the survey, sociodemographic information and information related to the respondents’ experience with cancer were collected. The remaining sections of the survey captured both quantitative and qualitative data on public views on the use of AI in cancer diagnosis and management and the use of data (deidentified personal health data and images of tissue) in the development of AI technologies for diagnostic pathology. Unless otherwise stated, responses to the closed questions were rated on a 5-point Likert scale measuring agreement (1=*strongly disagree*; 5=*strongly agree*).

To assess *awareness*, respondents were asked to rate their level of awareness of AI being developed for use in cancer diagnosis and treatment planning on a 5-point Likert scale (not at all to extremely). For *beliefs about the potential impact of AI*, respondents rated their level of agreement with the following 4 statements: “AI will help improve the diagnosis of cancer and treatment planning,” “AI will help reduce the risk of medical error or misdiagnosis,” “AI will result in less harm for patients,” and “AI will improve efficiency and performance in the NHS.” For *risks and benefits*, respondents could select any or all that applied from a list of potential risks (harm, error or misdiagnosis, lack of oversight or regulation, lack of accountability, loss of privacy, lack of personal interaction, or none) and benefits (more accurate decision-making, more efficiency, or money savings for the NHS; less risk of harm; less risk of error or misdiagnosis; more time freed up for staff to work on other tasks; or none) of using AI in cancer diagnosis and management.

To measure *support for the implementation of AI in diagnostic pathology*, respondents rated their level of agreement with the following 6 statements: “I support a diagnosis of cancer being made or ruled out by AI only,” “I support a diagnosis of cancer being made or ruled out by a pathologist with the assistance of AI,” “I support a diagnosis of cancer being made or ruled out by a pathologist only with no AI input,” “I support the diagnosis of biological ‘markers’ which can inform cancer treatment pathways by AI only,” “I support the diagnosis of biological ‘markers’ which can inform cancer treatment pathways being made by a pathologist with the assistance of AI,” and “I support the diagnosis of biological ‘markers’ which can inform cancer treatment pathways being made by a pathologist only with no AI input.”

For *support for the development of data-driven AI technologies for pathology*, respondents rated their level of agreement with the following 4 statements: “I support my deidentified personal health data being used in the development of AI technologies to diagnose and manage cancer by public bodies outside the NHS (eg, universities),” “I support my deidentified personal health data being used in the development of AI technologies to diagnose and manage cancer by private commercial research organizations outside the NHS,” “I support deidentified images of my tissue being used by public bodies outside the NHS (eg, universities),” and “I support deidentified images of my tissue being used by private commercial research organizations outside the NHS.”

To assess *trust in data security*, respondents rated their level of agreement with the following 6 statements: “I trust that my deidentified personal health data shared with public bodies outside the NHS (eg, universities) will not be used for purposes other than the development of AI technologies to diagnose and manage cancer,” “I trust that my deidentified personal health data shared with private commercial research organizations outside the NHS will not be used for purposes other than the development of AI technologies to diagnose and manage cancer,” “I trust that the NHS have effective security and privacy protocols for the sharing of personal health care data that will protect my anonymity,” “I trust that deidentified images of my tissue shared with public bodies outside the NHS (eg, universities) will not be used for purposes other than the development of AI technologies to diagnose and manage cancer,” “I trust that deidentified images of my tissue shared with private commercial research organizations outside the NHS will not be used for purposes other than the development of AI technologies to diagnose and manage cancer,” and “I trust that the NHS have effective security and privacy protocols for the sharing of image data that will protect my anonymity.”

Qualitative data were obtained from responses to the following open-ended questions: (1) “AI has the potential to improve the diagnosis and management of cancer, however patients and the public may have concerns about the development and application of AI in this context. What do you think would help yourself or others to support the use of AI technologies in cancer diagnosis and management? Please explain”; and (2) “AI has the potential to improve the diagnosis and management of cancer, however patients and the public may have concerns about the development and application of AI in this context. What do you think would help yourself or others to support the use of deidentified images of tissue and personal health data in the development of AI technologies to diagnose and manage cancer? Please explain.”

### Data Analysis

#### Quantitative Data Analysis

Descriptive statistics were calculated to assess public awareness of and attitudes toward the development and implementation of AI in pathology. Absolute numbers and percentages were reported. Unadjusted associations among sociodemographic characteristics, experiences of cancer, awareness, positive beliefs, number of risks and benefits identified, trust in data security, and support for the development and implementation of AI in pathology were assessed using an independent 2-tailed *t* test for continuous variables (awareness, positive beliefs, and number of benefits and risks identified) and the chi-square test for categorical variables. For continuous and categorical variables with >2 levels, unadjusted odds ratios (ORs) were obtained by separately fitting each variable against the binary support classification (univariate analyses). Statistical significance was determined as an α level of *P*<.05. Factors that were found to be related to support (using a less conservative threshold of *P*≤.10) were then entered into a multivariable logistic regression model using stepwise backward selection. Multivariable logistic regression examines the contribution of each variable in distinguishing between groups (those who did and did not support) while controlling for the other variables in the model and was used to assess the relative predictive ability of sociodemographic characteristics, experiences with cancer, awareness, beliefs, and attitudes in explaining support for (1) the use of AI in diagnostic pathology and (2) the development of data-driven AI technologies for diagnostic pathology.

#### Defining Quantitative Variables

Age was recoded into 4 categories (18-30, 31-45, 46-65, and ≥66 years), and gender was divided into 2 categories (men and women). The simplified ethnicity categories provided by Prolific (White, Black, Asian, mixed, and other) were used for statistical analyses beyond describing participant characteristics. Personal circumstances related to cancer were divided into 3 categories: those with personal experience (own diagnosis, close person or family member of someone diagnosed with cancer, or bereaved close person or family member of someone diagnosed with cancer), professional experience (professional or volunteer working with people diagnosed with cancer), or no direct experience. For the univariate and multivariable logistic regression analyses, composite scores were computed by summing the 6 items measuring trust in data security and the 4 items measuring positive beliefs about AI. New continuous variables were created for the number of potential risks and the number of potential benefits of AI identified by respondents. The outcome variable “support for implementation of AI in diagnostic pathology” was operationalized as strongly or somewhat agreeing with 4 statements related to the diagnosis of cancer and of biomarkers using AI only and by a pathologist with the assistance of AI. The outcome variable “support for development of AI technologies for pathology” was operationalized as strongly or somewhat agreeing with 4 statements related to the use of deidentified personal health data and images of tissue by public and private research bodies.

#### Qualitative Data Analysis

A total of 1472 free-text responses were received for the first open-ended question, and a total of 1457 responses were received for the second open-ended question. Reflexive thematic analysis was performed on the data derived from the open-ended questions. The 6-stage process described by Braun and Clarke [36] was carried out by CL. The process involved familiarization with the data through repeated reading of the responses; generating initial codes; identifying themes through code grouping; reviewing and refining the themes iteratively; and, finally, defining and naming the themes to construct a coherent narrative that reflects the patterns and meanings within the data. Reflexivity was integral to the rigor of the qualitative analysis, recognizing the influence of the research team’s backgrounds on interpretation. Thematic analysis was conducted by CL, who has expertise in pathology research and biobanking. The research team also included 2 health psychologists (JG and LGW) with no professional experience in pathology research or practice. Regular interdisciplinary discussions facilitated critical reflection, helping enhance the rigor of the analysis and ensure a balanced interpretation of the data. This process resulted in the identification of three themes: (1) *bringing the public along*, (2) *the human in the loop*, and (3) *more hard evidence needed*.

### Ethical Considerations

Ethics approval for this study was granted by the Queen’s University Belfast Faculty of Medicine, Health, and Life Sciences Research Ethics Committee (reference: MHLS 22_107). Relevant information such as background of the study, aims and objectives, and specific instructions about how to take part in the survey and what would be involved (eg, length of the survey and completion time) was clearly described on the front page of the survey website with a link to a detailed participant information sheet. Respondents were asked to indicate their consent to participate by using a checkbox. The survey was anonymous, no identifiable information was collected, and all data were released to the research team using a unique ID allocated by Qualtrics. Participants provided their age in years, and all other sociodemographic information was collected at a broad categorical level. A nominal monetary incentive was provided to respondents via Prolific in line with standardized rates (£2.80 [US $3.59]).

## Results

### Data Screening

An initial total of 1665 responses were received. One response was removed for not meeting eligibility criteria (ie, not a resident of the United Kingdom). In total, 0.54% (9/1665) of the responses were removed for not providing consent. The attrition rate was <1% (ie, 15/1665, 0.9% of the responses were removed for completing <50% of the survey items). The median completion time for this sample was 9.88 (IQR 5.84) minutes. A total of 3.06% (51/1665) of the responses were removed for having a completion time of less than half the median (ie, <4.93 min). In total, 1.92% (32/1665) of the responses were removed for failing 1 of 2 attention checks, and 1.74% (29/1665) were removed for evidencing straight-lining behavior on 2 matrix questions, indicative of low effort in responding. The final number of respondents in the dataset was 1518.

### Sample Characteristics

Age ranged from 18 to 82 years (mean 46.12, SD 15.68 years). A total of 51.32% (779/1518) of the sample were women; 47.63% (723/1518) were men; and 1.05% (16/1518) of the respondents identified as nonbinary, third gender, or other (eg, genderqueer) or preferred not to say. Most respondents were White (1328/1518, 87.48%), resided in England (1291/1518, 85.05%), were educated at the graduate level (992/1518, 65.35%), and had no religion (855/1518, 56.32%) or were Christian (537/1518, 35.38%).

When asked to describe their personal circumstances in relation to cancer, most respondents (910/1518, 59.95%) selected 1 response option, and 39.59% (601/1518) selected between 2 and 4 responses. A large proportion of respondents had experience of a close person or family member being diagnosed with cancer (617/1518, 40.65%), and 43.68% (663/1518) had experienced the death of a close person or family member to cancer. In total, 6.79% (103/1518) had been diagnosed with cancer themselves. A total of 4.68% (71/1518) of the respondents worked with people diagnosed with cancer (on a professional or voluntary basis), and 9.09% (138/1518) were health and social care professionals or academics with an interest in the subject of cancer.

### Quantitative Results

#### The UK Public’s Awareness of and Views on the Use of AI in Diagnostic Pathology

Awareness of AI being developed for use in diagnostic pathology was very low, with 43.21% (656/1518) of respondents being not at all aware and only 10.28% (156/1518) being moderately or extremely aware. However, the respondents held positive beliefs about the future implementation of AI in cancer diagnosis and management. Most respondents (1379/1518, 90.84%) somewhat or strongly agreed that AI will help improve the diagnosis of cancer and treatment planning, reduce the risk of medical error or misdiagnosis (1246/1518, 82.08%), improve efficiency and performance in the NHS (1339/1518, 88.21%), and result in less harm to patients (918/1518, 60.47%). Only a minority of respondents (96/1518, 6.32%) identified no risks of using AI in the diagnosis and management of cancer. A total of 10.14% (154/1518) of the respondents identified 5 or 6 risks. Risk of error was the most selected (1005/1518, 66.21%), followed by lack of accountability for decision-making (918/1518, 60.47%), lack of oversight or regulation (735/1518, 48.42%), and lack of personal interaction (737/1518, 48.55%). In total, 3.75% (57/1518) of the respondents identified no benefits of using AI in cancer diagnosis and management, and approximately one-third (556/1518, 36.63%) identified ≥4 benefits. More efficiency or money saving for the NHS were the most common benefit identified (1213/1518, 79.91%), followed by more time freed up for staff (1133/1518, 74.64%) and more accurate decision-making (1080/1518, 71.15%).

#### Support for the Use of AI in Diagnostic Pathology

##### Levels of Support for the Use of AI in Diagnostic Pathology

Most respondents (908/1518, 59.82%) did not support a cancer diagnosis being made or ruled out using AI only. A quarter of the respondents (370/1518, 24.37%) did. Similarly, most respondents (880/1518, 57.97%) supported a diagnosis being made by a pathologist only with no AI input, whereas 21.81% (331/1518) of the respondents did not. In relation to cancer, there was slightly more support for diagnosis of biomarkers being made using AI only (562/1518, 37.02%); however, almost half (694/1518, 45.72%) did not support this. Again, a large proportion of respondents (939/1518, 61.86%) supported diagnosis of biomarkers being made by a pathologist only with no AI input, whereas 18.97% (288/1518) did not. However, the vast majority supported a diagnosis of cancer (1478/1518, 97.36%) or of biomarkers (1479/1518, 97.43%) being made by a pathologist with the assistance of AI.

##### Predictors of Support for the Use of AI in Cancer Diagnosis and Management

###### Univariate Analyses

As shown in Table 1, there were gender differences in the support for the use of AI in cancer diagnosis and management. Specifically, being a man increased the probability of supporting implementation (OR 1.73, 95% CI 1.35-2.22). There were also age group differences—the odds of supporting implementation were 42% lower for those aged 18 to 30 years (OR 0.58, 95% CI 0.37-0.90). Identifying more potential risks of using AI in cancer diagnosis and management reduced the odds of supporting implementation (OR 0.70, 95% CI 0.64-0.77). However, identifying more potential benefits (OR 1.35, 95% CI 1.22-1.48), greater awareness (OR 1.40, 95% CI 1.25-1.57), more positive beliefs (OR 1.37, 95% CI 1.29-1.45), and higher trust (OR 1.09, 95% CI 1.06-1.12) increased the likelihood of supporting implementation.

**Table 1:** Sample characteristics and unadjusted associations with support for implementation of artificial intelligence (AI) in cancer diagnosis and management based on sociodemographic factors, experience with cancer, awareness, and attitudes toward using AI in diagnostic pathology (N=1518)^a^.

				Sample total		Low or no support (n=1188)		Support (n=330)		Unadjusted OR^b^ (95% CI)		β		*P* value	
**Sociodemographic factors, n (%)**															
	**UK nation**														.38
		England (reference)	1291 (85)		1011 (85.1)		280 (84.8)		—^c^		—		—		
		Scotland	122 (8)		100 (8.4)		22 (6.7)		0.79 (0.49-1.29)		−0.23		.35		
		Northern Ireland	39 (2.6)		27 (2.3)		12 (3.6)		1.60 (0.80-3.21)		0.47		.18		
		Wales	66 (4.3)		50 (4.2)		16 (4.8)		1.15 (0.65-2.06)		0.14		.62		
	**Gender**														<.001
		Men	723 (48.1)		529 (45.1)		194 (58.8)		1.73 (1.35-2.22)		0.55		<.001		
		Women (reference)	779 (51.9)		643 (54.9)		136 (41.2)		—		—		—		
	**Age group (y)**														.06
		18-30	341 (22.5)		282 (23.7)		59 (17.9)		0.58 (0.37-0.90)		−0.55		.01		
		31-45	413 (27.2)		326 (27.4)		87 (26.4)		0.74 (0.49-1.11)		−0.31		.15		
		46-65	591 (38.9)		453 (38.1)		138 (41.8)		0.84 (0.57-1.24)		−0.17		.38		
		≥66 (reference)	173 (11.4)		127 (10.7)		46 (13.9)		—		—		—		
	**Educational attainment**														.72
		Primary school	25 (1.6)		19 (1.6)		6 (1.8)		1.10 (0.44-2.79)		0.10		.84		
		Secondary school	501 (33)		398 (33.5)		103 (31.2)		0.90 (0.69-1.17)		−0.10		.45		
		Graduate level (reference)	992 (65.3)		771 (64.9)		221 (67)		—		—		—		
	**Experience with cancer**														.40
		Personal	643 (43.5)		512 (44.3)		131 (40.6)		0.85 (0.66-1.09)		−0.16		.21		
		Professional	18 (1.2)		15 (1.3)		3 (0.9)		0.67 (0.19-2.32)		−0.41		.52		
		No direct experience (reference)	818 (55.3)		629 (54.4)		189 (58.5)		—		—		—		
	**Religion**														.28
		No religion (reference)	855 (57.7)		681 (58.8)		174 (53.7)		—		—		—		
		Christian	537 (36.2)		405 (34.9)		132 (40.7)		1.27 (0.99-1.65)		0.24		.06		
		Buddhist	14 (0.9)		11 (0.9)		3 (0.9)		1.07 (0.29-3.97)		0.07		.92		
		Hindu	9 (0.6)		6 (0.5)		3 (0.9)		1.96 (0.48-7.90)		0.67		.35		
		Jewish	9 (0.6)		6 (0.5)		3 (0.9)		1.96 (0.48-7.90)		0.67		.35		
		Muslim	52 (3.5)		43 (3.7)		9 (2.8)		0.89 (0.39-1.71)		−0.20		.60		
		Sikh	7 (0.5)		7 (0.6)		0 (0)		0.00 (0.00-0.00)		−19.84		>.99		
	**Ethnicity**														.69
		Asian	102 (6.8)		85 (7.2)		17 (5.2)		0.70 (0.41-1.19)		−0.36		.19		
		Black	47 (3.1)		37 (3.1)		10 (3)		0.94 (0.46-1.92)		−0.06		.87		
		Mixed	20 (1.3)		17 (1.4)		3 (0.9)		0.61 (0.18-2.11)		−0.49		.44		
		White (reference)	1328 (87.9)		1032 (86.9)		296 (89.7)		—		—		—		
		Other	13 (0.9)		10 (0.8)		3 (0.9)		1.05 (0.29-3.83)		0.05		.95		
Awareness, mean (SD)				1.92 (1.01)		1.84 (0.97)		2.21 (1.10)		1.40 (1.25-1.57)		0.34		<.001	
Beliefs, mean (SD)				16.36 (2.64)		15.99 (2.69)		17.71 (1.93)		1.37 (1.29-1.45)		0.31		<.001	
Trust, mean (SD)				21.67 (5.76)		21.12 (5.82)		23.65 (5.09)		1.09 (1.06-1.12)		0.09		<.001	
Number of risks identified, mean (SD)				2.66 (1.45)		2.81 (1.44)		2.13 (1.33)		0.70 (0.64-0.77)		−0.35		<.001	
Number of benefits identified, mean (SD)				3.01 (1.34)		2.90 (1.33)		3.42 (1.28)		1.35 (1.22-1.48)		0.30		<.001	

^a^Unadjusted associations were assessed using chi-square tests for categorical variables, univariate logistic regression for variables with >2 levels, and independent *t* tests for continuous variables.

^b^OR: odds ratio.

^c^Not applicable for reference categories.

###### Multivariable Logistic Regression

The 7 variables that were significant at the α level of <.10 in the univariate analyses were entered into multivariable logistic regression. The final model with adjusted ORs (aORs) with 95% CIs for the predictors is shown in Table 2. Holding all other predictor variables constant, the odds of supporting the implementation of AI in cancer diagnosis and management were 34% greater for men than for women (aOR 1.34, 95% CI 1.02-1.75). The odds of supporting implementation increased by 25% (aOR 1.25, 95% CI 1.10-1.42) for every 1-unit increase in awareness of AI and by 27% for every 1-unit increase in positive beliefs about the potential impact of AI (aOR 1.27, 95% CI 1.20-1.36). The likelihood of supporting implementation increased by 4% for every 1-unit increase in trust in data security (aOR 1.04, 95% CI 1.01-1.07) and decreased by 20% (aOR 0.80, 95% CI 0.73-0.89) for every 1-unit increase in the number of identified risks.

**Table 2:** Multivariable logistic regression analysis of factors predicting support for implementation of artificial intelligence in cancer diagnosis and management.

		β^a^	aOR^b^ (95% CI)	*P* value
Constant		−1.27	0.282^c^	<.001
Men		0.29	1.34 (1.02-1.75)	.04
**Age group (y)**				
	18-30	−0.11	0.89 (0.55-1.43)	.63
	31-45	−0.13	0.87 (0.56-1.36)	.55
	46-65	0.08	1.08 (0.71-1.65)	.71
	≥66 (reference)	—^d^	—	—
Awareness		0.22	1.25 (1.10-1.42)	.001
Beliefs		0.24	1.27 (1.20-1.36)	<.001
Trust		0.04	1.04 (1.01-1.07)	.008
Number of risks identified		−0.22	0.80 (0.73-0.89)	<.001
Number of benefits identified		0.06	1.06 (0.95-1.19)	.30

^a^Regression coefficient.

^b^aOR: adjusted odds ratio.

^c^Not applicable for constant.

^d^Not applicable for reference categories.

#### Support for the Use of Deidentified Personal Health Data and Images of Tissue in the Development of AI Technologies

##### Levels of Support

There was support for the use of deidentified personal health data (1275/1518, 83.99% agreed or strongly agreed) and images of tissue (1326/1518, 87.35%) in the development of AI technologies by public bodies outside the NHS (eg, universities). Support was lower for the use of deidentified personal health data (869/1518, 57.25%) and images of tissue (958/1518, 63.11%) by private commercial research organizations. The difference in support for public versus private bodies was statistically significant for both personal health data (χ^2^_1_=308.4; *P*<.001) and images of tissue (χ^2^_1_=310.9; *P*<.001).

##### Trust in Data Security

Despite the high levels of support for the development of data-driven AI technologies by public bodies outside the NHS, there were comparatively lower levels of trust that deidentified personal health data (1070/1518, 70.49%) and images of tissue (1147/1518, 75.56%) would not be used for other purposes by such public bodies. There was less trust again that deidentified personal health data (688/1518, 45.32%) and images of tissue (778/1518, 51.25%) would not be used for other purposes by commercial organizations. The difference in trust for public versus private bodies was statistically significant for both personal health data (χ^2^_1_=405.0; *P*<.001) and images of tissue (χ^2^_1_=413.3; *P*<.001). Nevertheless, there was very high trust that the NHS security and privacy protocols for the sharing of personal health care data (1068/1518, 70.36%) and images of tissue (1098/1518, 72.33%) would protect people’s anonymity.

##### Predictors of Support for the Development of Data-Driven AI Technologies for Cancer Diagnosis and Management

###### Overview

There were significant age group differences in support for the development of AI in cancer diagnosis and management (Table 3). The odds of supporting development were significantly lower for those aged 18 to 30 years (OR 0.45, 95% CI 0.31-0.66) and 31 to 45 years (OR 0.54, 95% CI 0.38-0.78). Working with people with cancer reduced the odds of supporting the development of AI for diagnostic pathology (OR 0.35, 95% CI 0.12-0.98). There were differences in support based on religious affiliation. With respect to those with no religion, the odds of supporting development were 40% higher for Christian people (OR 1.40, 95% CI 1.12-1.74) and 61% lower for Muslim people (OR 0.39, 95% CI 0.21-0.72). Compared to White respondents, those of mixed (OR 0.43, 95% CI 0.17-1.08), Asian (OR 0.33, 95% CI 0.21-0.52), Black (OR 0.50, 95% CI 0.27-0.90), or another ethnicity (OR 0.15, 95% CI 0.03-0.66) had a lower probability of supporting development. Respondents who identified more potential risks of using AI in cancer diagnosis and management had lower odds of supporting development (OR 0.68, 95% CI 0.63-0.74). Those who identified more potential benefits (OR 1.46, 95% CI 1.34-1.58) and had more positive beliefs (OR 1.21, 95% CI 1.16-1.27) and more trust in data security (OR 1.43, 95% CI 1.36-1.46) had an increased likelihood of supporting development.

**Table 3:** Sample characteristics and unadjusted associations with support for the development of data-driven artificial intelligence (AI) in cancer diagnosis and management based on sociodemographic factors, experience with cancer, awareness, and attitudes toward AI in diagnostic pathology (N=1518)^a^.

				Sample total		Low or no support (n=722)		Support (n=796)		Unadjusted OR^b^ (95% CI)		β		*P* value	
**Sociodemographic factors, n (%)**															
	**UK nation**														.69
		England (reference)	1291 (85)		608 (84.2)		683 (85.8)		—^c^		—		—		
		Scotland	122 (8)		59 (8.2)		63 (7.9)		0.95 (0.66-1.38)		−0.05		.79		
		Northern Ireland	39 (2.6)		19 (2.6)		20 (2.5)		0.94 (0.50-1.77)		−0.07		.84		
		Wales	66 (4.3)		36 (5)		30 (3.8)		0.74 (0.45-1.22)		−0.30		.24		
	**Gender**														.62
		Men	723 (48.1)		337 (47.5)		386 (48.7)		1.05 (0.89-1.29)		0.05		.62		
		Women (reference)	779 (51.9)		373 (52.5)		406 (51.3)		—		—		—		
	**Age group (years)**														<.001
		18-30	341 (22.5)		197 (27.3)		144 (18.1)		0.45 (0.31-0.66)		−0.80		<.001		
		31-45	413 (27.2)		220 (30.5)		193 (24.2)		0.54 (0.38-0.78)		−0.61		<.001		
		46-65	591 (38.9)		239 (33.1)		352 (44.2)		0.91 (0.64-1.29)		−0.10		.59		
		≥66 (reference)	173 (11.4)		66 (9.1)		107 (13.4)		—		—		—		
	**Educational attainment**														.17
		Primary school	25 (1.6)		10 (1.4)		15 (1.9)		1.46 (0.65-3.28)		0.38		.36		
		Secondary school	501 (33)		223 (30.9)		278 (34.9)		1.21 (0.98-1.50)		0.19		.08		
		Graduate level (reference)	992 (65.3)		489 (67.7)		503 (63.2)		—		—		—		
	**Experience with cancer**														.10
		Personal	643 (43.5)		299 (42.8)		344 (44.1)		1.03 (0.84-1.27)		0.03		.76		
		Professional	18 (1.2)		13 (1.9)		5 (0.6)		0.35 (0.12-0.98)		−1.06		.045		
		No direct experience (reference)	818 (55.3)		387 (55.4)		431 (55.3)		—		—		—		
	**Religion**														<.001
		No religion (reference)	855 (57.7)		419 (59.9)		436 (55.7)		—		—		—		
		Christian	537 (36.2)		219 (31.3)		318 (40.6)		1.40 (1.12-1.74)		0.33		.003		
		Buddhist	14 (0.9)		9 (1.3)		5 (0.6)		0.53 (0.18-1.61)		−0.63		.26		
		Hindu	9 (0.6)		6 (0.9)		3 (0.4)		0.48 (0.12-1.93)		−0.73		.30		
		Jewish	9 (0.6)		5 (0.7)		4 (0.5)		0.77 (0.21-2.89)		−0.26		.70		
		Muslim	52 (3.5)		37 (5.3)		15 (1.9)		0.39 (0.21-0.72)		−0.94		.003		
		Sikh	7 (0.5)		5 (0.7)		2 (0.3)		0.38 (0.07-1.99)		−0.96		.26		
	**Ethnicity**														<.001
		Asian	102 (6.8)		72 (10.1)		30 (3.8)		0.33 (0.21-0.52)		−1.10		<.001		
		Black	47 (3.1)		29 (4.1)		18 (2.3)		0.50 (0.27-0.90)		−0.70		.02		
		Mixed	20 (1.3)		13 (1.8)		7 (0.9)		0.43 (0.17-1.08)		−0.85		.07		
		White (reference)	1328 (87.9)		589 (82.5)		739 (92.8)		—		—		—		
		Other	13 (0.9)		11 (1.5)		2 (0.3)		0.15 (0.03-.66)		−1.93		.01		
Awareness, mean (SD)				1.92 (1.01)		1.90 (1.03)		1.94 (1.00)		1.04 (0.94-1.15)		0.04		.42	
Beliefs, mean (SD)				16.36 (2.64)		15.71 (2.68)		16.96 (2.46)		1.21 (1.16-1.27)		0.19		<.001	
Trust, mean (SD)				21.67 (5.76)		17.99 (5.17)		24.99 (3.98)		1.43 (1.36-1.46)		0.35		<.001	
Number of risks, mean (SD)				2.66 (1.45)		3.05 (1.43)		2.30 (1.37)		0.68 (0.63-0.74)		−0.38		<.001	

^a^Unadjusted associations were assessed using chi-square tests for categorical variables, univariate logistic regression for variables with >2 levels, and independent *t* tests for continuous variables.

^b^OR: odds ratio.

^c^Not applicable for reference categories.

###### Multivariable Logistic Regression

The 8 variables that were significant at the α level of <0.10 in the univariate analyses were entered into multivariable logistic regression. The final model with aORs with 95% CIs for the predictors is shown in Table 4. Holding all other variables constant, the odds of supporting the development of data-driven AI technologies were 59% lower for people of Asian ethnicity (aOR 0.41, 95% CI 0.23-0.71) and 86% lower for people of another (non-White, non-Black, or not mixed) ethnicity (aOR 0.14, 95% CI 0.02-0.79). The odds of supporting the development of data-driven AI technologies increased by 42% for every 1-unit increase in trust (aOR 1.42, 95% CI 1.36-1.47) and increased by 32% for every 1-unit increase in the number of benefits identified (aOR 1.32, 95% CI 1.19-1.47).

**Table 4:** Multivariable logistic regression analysis of factors predicting support for the development of data-driven artificial intelligence technologies in diagnostic pathology.

		β^a^	aOR^b^ (95% CI)	*P* value
Constant		0.12	1.13^c^	.02
**Age group (years)**				
	18-30	−0.37	0.69 (0.42-1.14)	.63
	31-45	−0.27	0.77 (0.47-1.90)	.55
	46-65	0.18	1.20 (0.75-1.90)	.70
	≥66 (reference)	—^d^	—	—
**Ethnicity**				
	Asian	−0.89	0.41 (0.23-0.71)	.002
	Black	−0.41	0.67 (0.29-1.53)	.34
	Mixed	−0.47	0.62 (0.19-2.04)	.43
	White (reference)	—	—	—
	Other	−1.98	0.14 (0.02-0.79)	.03
**Religion**				
	No religion (reference)	—	—	—
	Christian	−0.04	0.96 (0.71-1.31)	.81
	Buddhist	−0.48	0.62 (0.11-3.61)	.60
	Hindu	−0.63	0.53 (0.07-3.81)	.53
	Jewish	1.12	3.05 (0.41-22.97)	.28
	Muslim	−0.38	0.68 (0.25-1.85)	.45
	Sikh	−1.01	0.37 (0.04-3.06)	.35
Beliefs		0.01	1.00 (0.94-1.07)	.96
Trust		0.35	1.42 (1.36-1.47)	<.001
Number of risks		−0.09	0.92 (0.82-1.02)	.12
Number of benefits		0.28	1.32 (1.19-1.47)	<.001

^a^Regression coefficient.

^b^aOR: adjusted odds ratio.

^c^Not applicable for constant.

^d^Not applicable for reference categories.

### Qualitative Results

#### Theme 1: Bringing the Public Along

Confirming the quantitative findings, when asked about what would help increase support for the use of AI in pathology, respondents frequently cited the need for greater public knowledge about its use. This was emphasized in relation to both increasing basic awareness of what AI is and supporting the public in understanding the inner workings of AI in pathology through accessible yet detailed explanations or demonstrations to “see the AI in action” (man; aged 62 years):

The general public has been hearing about AI now for many years but I still believe there is a lot of confusion about the genuine benefits that it can bring and still feels like a “concept” rather than technology that is with us day to day now.

Man; aged 42 years

I think it needs to be explained and the public made more aware of how it works.

Woman; aged 54 years

In addition to the discussion on improving public knowledge, there was an emphasis on the importance of “transparency” and “openness,” including information on the extent of human oversight within the process, with common concerns about undetected “glitches” or “malfunctions” in AI systems. A greater understanding of AI was linked to fostering “trust” in rather than “fear” of the technology:

Better transparency of the process and how it works, and how much doctors rely on the technology.

Man; aged 55 years

I think it would need to be better understood what AI could do and how this could help. If people don’t understand then they are more likely to be scared.

Woman; aged 31 years

Risk management-how it will be ensured that we don’t rely on AI only to miss fatal diagnoses.

Woman; aged 26 years

The respondents suggested various ways in which awareness of such technologies could be achieved. These included public awareness-raising campaigns via advertising and social media. The benefits of sharing “real-world” patient stories were also discussed to illustrate the individual patient benefits of the use of AI:

By talking about it more through all sorts of different media, especially by commissioning a segment on TV, and a massive social media campaign. Talk people through exactly how it works so that we gain a better understanding of it all.

Woman; aged 42 years

Case studies demonstrating how the application of artificial intelligence technologies have generated value.

Man; aged 24 years

Examples of it being used either alone or with human assistance to successfully diagnose and/or manage cancer in a patient.

Woman; aged 44 years

In response to the open question about support for the use of deidentified tissues and data in the development of AI technologies, the desire for more reassurance was commonly cited by respondents. The potential misuse of deidentified data was the most frequently mentioned barrier for most respondents when it came to support for the use of AI. Many respondents expressed a need for clear communication on how their data are used by AI, including how their data are managed, stored, and protected:

If the whole process was explained in easy to understand terms from collecting their data to how their data is used, why it’s stored, how AI uses this de-identified data to improve its own capabilities.

Man; age 22 years

A much clearer understanding of what it involves. People don’t want to be blinded by science at the time of diagnosis.

Woman; aged 62 years

Many respondents expressed caution or reticence when it came to support for their data being shared with commercial entities. Concerns about data protection were nearly entirely focused on commercial entities, with a higher level of trust that the NHS and public bodies (such as universities) would act responsibly and ensure patient benefit from the data:

I personally do not have a problem with these images being shared with the NHS as they are a public body. I do have a problem with this data being shared with private organisations that would then charge the NHS for these services. Being transparent about the use of the data is important to me and others.

Man; aged 51 years

The use of this information can only be for the greater good.

Man; aged 64 years

Many respondents expressed a need for robust oversight measures to be in place to protect their personal information. The potential for misuse of data was frequently linked with the use of data for financial gain, with various high-profile examples given in which personal data had been misused. While many respondents referred to data protection legislation, many also requested close monitoring of the use of data and tissues by non-NHS bodies. It was also suggested that organizations should be held accountable if data are misused:

Explain the security measures that are put in place to make sure that the data remains anonymous. Make sure there are independent audits of the security measures.

Man; aged 41 years

Strict guidelines with respect to data protection. Large fines with appropriate legal actions against bodies that use the images inappropriately.

Man; aged 25 years

Stronger consequences for organisations that breach data protection policies. Increased transparency and vetting by independent sources with regards to these organisations.

Woman; aged 27 years

#### Theme 2: The Human in the Loop

Responses to the open question on support for the use of AI in cancer diagnosis and management largely indicated that respondents would be supportive of “assistance” and “support” from AI provided there was human oversight of the technology in the form of checks and balances. There was concern over the potential for undetected errors with the use of AI alone, particularly with novel use of AI. Responses included the following:

Ensuring that there is still a human face to it.

Man; aged 32 years

In the early days of use, need to use both AI and pathologists—need to check the validity of diagnosis.

Woman; aged 64 years

Conversely, respondents also frequently cited the potential for human error in diagnoses, with AI likened to obtaining a second opinion from another clinician. Many respondents highlighted the potential for combined AI and human input to reduce error on both parts:

If it was not used alone, but with human supervision. Both humans and AI can make mistakes—by working together this can be reduced.

Woman; aged 23 years

There was a frequently mentioned concern over the extent to which the use of AI reduces human control over the decision-making process. The respondents emphasized the importance of AI being used as a tool “by” physicians rather than to “replace” physicians, with clinical experience ultimately prioritized. The potential for AI to improve the efficiency of the decision-making process was widely recognized:

So long as the final decision is always made by a qualified human, I see no problem in using AI as a shortcut to get to the point where the decision is made.

Woman; aged 74 years

I think folk’s main concern would be the idea of a system making decisions about them in an opaque way they had no input on. If it’s explained that the AI is used to support a physician rather than supplant them, I think folk would be considerably reassured.

Man; aged 40 years

#### Theme 3: More Hard Evidence Needed

The respondents recognized the potential for AI to provide efficiency savings for the health care system and the potential for this to directly impact patient care. However, to enhance public support, respondents also reported a need for “proof” of the success of AI technologies. There was the overall impression of a lack of research in this area and the need to build the evidence base:

A fully funded clinical trial, that shows explicitly that AI is effective, even more effective than the current system.

Man; aged 22 years

Respondents felt that high-quality research to demonstrate the accuracy of AI against human-only input in reducing the number of missed diagnoses or demonstrating the efficiency of AI would be the most convincing for public support:

Studies which could demonstrate the effectiveness of AI in improving diagnosis and treatment outcomes when compared with human-only diagnosis and treatment.

Man; aged 48 years

A series of research studies which showed how effective AI can be to reduce waiting times for diagnosis and treatment.

Woman; aged 61 years

Overall, the burden of proof is on AI, and respondents called for evidence that AI is not only equivalent to a human in performance but surpasses a human in accuracy and reliability of diagnosis. Respondents highlighted a desire for high-level statistics on the overall accuracy of AI:

Success stories where AI has achieved what a human cannot.

Woman; aged 32 years

If it is totally error free, I would be in favour of AI.

Man; aged 32 years

Respondents also stated the need for reassurance in the form of evidence that data and tissues would indeed be deidentified before transfer outside the NHS, for example, through detailing the process through which the data would be prepared and handled:

Evidence that data is held and transmitted securely.

Man; aged 66 years

I would need to understand what the procedure for de-identification actually is, and be convinced that it is effective.

Man; aged 67 years

## Discussion

### Principal Findings

This study sought to examine the UK public’s awareness of and attitudes toward the use of AI in pathology research and practice and ascertain predictors of support for its implementation in clinical practice and for the use of health data and tissue images in its development. We found that awareness of AI being developed for use in pathology was low, yet respondents were conditionally supportive of its use in the diagnosis and management of cancer if human oversight was maintained. Respondents were also supportive of their data and tissue images being used in the development of such technologies by public bodies; however, there was less support for use by commercial bodies. To our knowledge, this is the only nationally representative study on public opinion on AI in pathology, a field in which AI has been regarded as the “third revolution” [37].

### Comparison to Prior Work

Awareness of AI being developed for use in pathology was very low, which was not overly surprising. While awareness of AI in general is increasing, a 2023 nationally representative survey of public attitudes toward AI in Great Britain reported that people were less aware of the use of AI in health compared to the use of AI in other applications such as facial recognition, driverless cars, and targeted consumer advertising [31]. In this study, qualitative data indicated a broad appetite for increased awareness and understanding of how AI systems could be used in cancer diagnosis and management. Suggestions from respondents on how to deliver this included social media campaigns and information disseminated via leaflets, videos, and television media using real-world examples of how AI can benefit their care. Despite low awareness, the respondents in this study held positive beliefs about the potential of AI to improve patient outcomes and health care system efficiency. Accordingly, the most frequently identified benefits of AI in pathology practice were maximization of NHS resources and more accurate decision-making. However, the most frequently identified risks were error, lack of accountability, and lack of oversight. These risks and benefits of AI in pathology are in keeping with those reported in studies investigating the use of AI in other medical specialties such as radiology [38] and dermatology [39]. Almost all respondents supported a diagnosis of cancer or an assessment of biomarkers being made by a pathologist with the assistance of AI, but there was no widespread support for diagnosis using AI only. This was also evident from the qualitative analysis, with many respondents indicating that some form of human oversight would help increase their support for the use of AI in pathology practice. This position is consistent with previous literature suggesting that the acceptance of AI in health care is overwhelmingly based on its use as a tool to support clinician decision-making rather than as a replacement [30,40].

There was strong support for the use of deidentified personal health data and images of tissue in the development of AI technologies for pathology by public bodies. This is reassuring given the potential impact of controversial failed NHS data-sharing projects such as care.data [41] and the General Practice Data for Planning and Research program [42] on public opinion. There was slightly less support for the use of health data and images by private bodies (vs public bodies), confirming previous research findings indicating a level of concern among the public when it comes to private or commercial organizations obtaining access to health data [16,43]. Although there were high levels of trust in NHS security and privacy protocols, there was some concern that data would be used for other purposes by both public and commercial organizations, with qualitative analysis highlighting that transparency and openness in how data are shared and governed would help increase support for the use of data in the development of AI health systems. As outlined in a recent UK Government review into the use of health care data in research [44], our findings reinforce the need for candid and continuous engagement with the public about data sharing, particularly when this involves commercial organizations.

Identifying more benefits of AI in pathology practice increased the odds of supporting the development of AI in pathology research, whereas identifying more risks decreased the odds of supporting implementation of AI in pathology practice. Greater awareness of and more positive beliefs about AI increased the likelihood of supporting implementation, and men were more likely to support the use of AI in cancer diagnosis and management relative to women. This is a finding that is common to the wider AI literature, in which women appear to be more cautious in their opinions on AI in health care than men. Yakar et al [45] surveyed 2411 members of the public in the Netherlands in 2022 regarding their views on AI in medicine with specific emphasis on dermatology, radiology, and robotic surgery and reported more trust in AI among their male respondents than among their female respondents. Similarly, a large web-based survey (N=11,004) of Americans’ views on AI in health and medicine conducted by the Pew Research Center in 2022 concluded that men were more open to the use of AI in their health care than women [46]. Although our data cannot explain why women or people from a non-White background are less likely to support AI in pathology, our findings correspond with the existing evidence of a notable lack of support for AI in health care among these populations. Given the evidence of gender inequities in the ability of AI tools to detect liver disease [47] and the concern that datasets used to train AI algorithms will exacerbate ethnic health care inequalities if they are ethnically unbalanced or biased [48], it is imperative that those involved in the development and implementation of AI technologies in pathology engage meaningfully with these populations to better understand their concerns.

Fundamentally, AI has the potential to revolutionize health care. Although the number of AI systems currently being used in health care in the United Kingdom remains relatively low, this number will undoubtedly increase within the next decade given the rate of their development along with committed investment from the government to embrace and deploy AI technologies in the NHS [49]. However, the acceptability of AI and, ultimately, its success will depend on public confidence and trust in its development and implementation [50]. While the Academy of Medical Royal Colleges recommends early and ongoing public engagement to maximize the impact and acceptability of AI in health care [34], our findings suggest that aspects of this may have been left wanting to date, particularly in the field of pathology, where AI technologies are being developed at such a rapid pace.

Presently, the United Kingdom lacks specific legislation governing the use of AI, opting instead for a “proportionate and light touch” framework that will provide a set of principles for regulatory authorities to interpret and implement according to the requirements of their own sector [51]. This approach, deemed proinnovative, aligns with the rapidly evolving AI landscape. However, our research underscores that public support for the use of AI in pathology is contingent on maintaining human oversight in clinical decision-making; therefore, regulatory bodies and stakeholders who govern the application of AI technologies not only in pathology but also in the wider health care milieu recognize that any shift from this position may undermine public confidence and trust. To ensure support for the use of AI in pathology, stakeholders should prioritize initiatives fostering awareness and promoting positive beliefs about AI. Awareness campaigns should adopt a balanced perspective that acknowledges potential risks while concurrently emphasizing the benefits. Stakeholders must inform the public of the evidence supporting the use of AI in health care while reassuring the public regarding robust data security measures in place in its development. Simultaneously, efforts should be made to bridge gender disparities in support, and specific strategies should be developed to address the lower support among individuals from diverse backgrounds, ensuring inclusivity in AI discussions and decision-making. Through a balanced approach encompassing transparency, engagement, and targeted education, policy makers can cultivate a supportive and informed public stance regarding the responsible implementation and development of AI in health care, aligning with the evolving regulatory landscape.

### Strengths and Limitations

This study is novel in that it is the first to examine public awareness of and opinions on the use of AI in pathology research and practice with a nationally representative UK sample. However, there are several limitations to acknowledge. First, selection bias was a possible limitation in this study due to the web-based nature of the survey as those without an internet connection or some level of digital literacy had restricted access. Second, a known limitation of using a panel survey is the risk to data integrity due to inattentive responses [52]. Although this risk was mitigated in this study by using attention checks, screener validation questions, and a Completely Automated Public Turing Test to Tell Computers and Humans Apart, along with the removal of responses indicative of low effort in responding, there remains potential for low-quality responses due to the uncontrolled nature of the survey. Third, it was also noted that a large proportion (992/1518, 65.35%) of the respondents in the survey were educated at the graduate level; this may have implications for the findings given claims that attitudes on societal and political issues change incrementally with educational level [53]. Finally, the survey questions used in this study were developed by the research team based on the study objectives and the existing literature. However, to the best of our knowledge, no validated standardized questionnaire on the development and implementation of AI in pathology or even health care was available when this study was conducted.

### Future Directions

Future work to validate a tool to longitudinally assess public knowledge of and trust in AI in health care would be of value, in line with AI being implemented more widely in many aspects of health care delivery. There is also a need to further explore the gender and ethnic disparities that were uncovered in this study to recognize the specific concerns of women and those from a non-White background. Given the suggested variances between countries in their level of trust in AI in general [54], further research representative of wider geographical areas would be of merit.

### Conclusions

Our findings showed a low level of awareness of the use of AI in cancer diagnosis and management. Respondents were positive about the potential of AI and indicated a high level of support for its use, albeit conditional on the fact that human oversight of the process is maintained. Analysis of qualitative data on support for AI reinforced the need for transparency and regulation regarding the use of images and personal health care data for research purposes. It also largely indicated that respondents would be supportive of assistance from AI in their cancer diagnosis and management provided there was human oversight of the process and evidence of AI’s ability. Notably, our findings demonstrated that challenges remain in addressing the concerns specifically of women and apprehension remains about the access to and use of health care data by commercial organizations.

The findings of this study have important implications for all stakeholders involved in the development and implementation of AI in pathology research and practice. They indicate the need for a balanced approach to the process, one that comprises openness, engagement, and education to cultivate a supportive and informed public. It is imperative that AI developers, researchers, and organizations deploying AI applications take this balanced approach into account to align with the expectations and concerns of the public regarding such technologies.

## Appendix

Multimedia Appendix 1 Web-based survey questions.

## Abbreviations

AI: artificial intelligence
aOR: adjusted odds ratio
NHS: National Health Service
OR: odds ratio
WSI: whole slide image

## Data Availability

The datasets generated of analyzed during this study are available from the corresponding author on reasonable request.
