# Supplementary material for: Public Awareness of and Attitudes Toward the Use of AI in Pathology Research and Practice: Mixed Methods Study
Source: J Med Internet Res. 2025 Apr 2;27:e59591. doi: 10.2196/59591 (PMC12004022; doi:10.2196/59591)
Supplement: Multimedia Appendix 1 [file jmir_v27i1e59591_app1.pdf]

Prolific ID

What is your Prolific ID?
Please note that this response should auto-fill with the correct ID

${e://Field/PROLIFIC_PID}

Screener Validation

I am currently living in the United Kingdom

- Yes
- No

I am over 18 years of age

- Yes
- No

Study Information

Public awareness and attitudes toward the use of Artificial Intelligence in Cancer Diagnosis and Management Background

Background

We, a team of researchers from Queen's University Belfast and the University of Galway, want to find out about people's awareness and attitudes towards the use of artificial intelligence (AI) in cancer diagnosis and management. You do not need to have any prior knowledge of AI or previous experience of cancer to take part.

Taking part in this survey is voluntary and there are no right or wrong answers.

We hope that the findings will help us to understand the extent of awareness of AI and peoples' views on the potential use of AI technology in their healthcare. We will publish the results of this study in the form of general reports and academic papers. Your name will not be collected or used anywhere.

When using the internet, there can be a risk to your privacy, confidentiality and/or anonymity. We have taken the precaution of using the Qualtrics Online Survey Tool as it is known to be safe to use to minimise this risk.

We will use and manage all data in line with the General Data Protection Regulation (2018) currently used in the UK. This study has been approved by the Research Ethics Committee of the Faculty of Medicine, Health and Life Sciences, Queen's University Belfast.

We would like to thank you in advance for your help and time. If you have any questions, please contact Dr Claire Lewis, Honorary Lecturer at the School of Medicine, Dentistry, and Biomedical Sciences at Queen's University Belfast on claire.lewis@qub.ac.uk.

Instructions

- It should take about 15 minutes to complete.
- Please add any comments in the blank boxes that you feel are relevant or important.
- If you feel upset while filling out this survey, you can complete the survey in more than one sitting if you like. To do this you can exit the survey browser and resume your progress later (up to one week), so long as you return on the same browser where you started the survey.
- If you wish to end the survey while it is in progress, you can simply exit the page.
- Due to the anonymous nature of the study, you will not have the opportunity to withdraw your data after submitting/exiting the survey.
- You can browse the survey and change answers up to the point when you click the finish button to register your submission.

Select the Yes option for more information about the study

○ Yes, I would like more information

Participant Information Sheet

Public awareness and attitudes toward the use of Artificial Intelligence in Cancer Diagnosis and Management

What is the purpose of the research?

We, a team of researchers from Queen's University Belfast and the University of Galway, want to find out about people's awareness and attitudes towards the use of artificial intelligence (AI) in cancer diagnosis and management. You do not need to have any prior knowledge of AI or previous experience of cancer to take part.

Why am I being asked to participate?

You have been invited to take part in this study as a member of the public. We define a member of the public as individual people currently living in the UK and who are 18 years old or above. It is up to you to decide to join the study. We will describe the study and if you agree to take part, we will ask you to select a box on the online form to provide your consent to take part.

What am I being asked to do?

If you agree to take part in the research, it will involve completing an online survey. The survey will take approximately 15 minutes to complete. Please add any comments in the blank boxes that you feel are relevant or important. You can complete the survey in more than one sitting if you like. To do this you can exit the survey browser and resume your progress later (up to one week), so long as you return on the same browser where you started the survey. You can browse the survey and change answers up to the point when you click the final

button to register your submission.

Do I have to take part?

It is your decision whether to take part in the study. You are free to withdraw from the study at any point during data collection by exiting the survey on your browser. Due to the anonymous nature of the study, you will not have the opportunity to withdraw your data after submitting the survey.

What happens with my information?

When using the internet, there can be a risk to your privacy, confidentiality and/or anonymity. We have taken the precaution of using the Qualtrics Online Survey Tool as it is known to be safe to use to minimise this risk. We will not collect your name or any other information which may identify you. As a result, the research team will not be able to and will not contact you. Any information that you do provide will be kept securely by those named on the research team. Any results which are published within the scientific community will not include any information which could identify you. You are also agreeing that we can include your responses and anonymised extracts of the survey in future reports, in professional journals, at professional conferences, in educational settings and for future research.

Are there any risks or benefits to taking part?

This research has been approved by the Research Ethics Committee of the Faculty of Medicine, Health and Life Sciences, Queen's University Belfast. The study is low risk, with no reports of harm from similar research conducted in the past. If you feel upset while filling out this questionnaire you can complete it in more than one sitting if

you like. To do this you can exit the survey browser and resume your progress later (up to one week), so long as you return on the same browser where you started the survey. You can browse the survey and change answers up to the point when you click the finish button to register your submission. If you wish to end the survey while it is in progress, you can exit the survey on your browser. Due to the anonymous nature of the study, you will not have the opportunity to withdraw your data after submitting/exiting the survey. We would hope that participation in the research is a positive experience, allowing you to reflect upon your own awareness and attitudes towards the use of AI generally and as applied to health.

What if there is a problem?

If you experience any kind of problem, you can contact Dr Claire Lewis, Honorary Lecturer at the School of Medicine, Dentistry, and Biomedical Sciences at Queen's University Belfast on claire.lewis@qub.ac.uk
If you have a complaint, you can contact the Head of QUB Research Governance, Ethics, and Integrity on researchgovernance@qub.ac.uk

Many thanks for your interest in our research.
The Research Team

Consent

By agreeing to participate in this survey, you state that:

- you currently live in the UK

- you have read and understood the information above and
- you are aged 18 or over.

You are also agreeing that we can include your responses and anonymised extracts of the survey in future reports. Extracts may also be published in professional journals, presented at professional conferences and in educational settings. The information provided may be used for producing reports and academic papers and for future research.

Do you agree to participate in this survey?

- Yes
- No

Captcha

Before you proceed to the survey, please complete the captcha below

I'm not a robot

reCAPTCHA
Privacy - Terms

About you

Which of the following statements describe your personal circumstances (select all that apply)

- ☐ I have previously received a cancer diagnosis
- ☐ I am a carer/family member/partner/spouse/friend/loved one of someone who has previously received a cancer diagnosis
- ☐ I am a bereaved carer/family member/partner/spouse/friend who has lost a loved one to cancer
- ☐ I am a professional working with people who have been diagnosed with cancer
- ☐ I am a volunteer working with people who have been diagnosed with cancer
- ☐ I am a health/social care professional (clinical)
- ☐ I am a health/social care professional (non-clinical)
- ☐ I am a health researcher/an academic who has an interest in the subject
- ☐ I am a member of the public who has an interest in the subject
- ☐ Other (please write in) ______

What is your age in years

______

What is your gender?

- ○ Man
- ○ Woman
- ○ Non-binary / third gender
- ○ Prefer not to say
- ○

- Other (please write in) ____________

Where in the UK do you live?

- England
- Scotland
- Northern Ireland
- Wales

Please select one of the following options that best describes your religious belonging

- No Religion
- Christian
- Buddhist
- Hindu
- Jewish
- Muslim
- Sikh
- Prefer not to say
- Other (please write in) ____________

There are 14 months in a year. This is an attention check.

- Definitely true
- Probably false
- Probably true
- Definitely false

Please select one of the following options that best describes your ethnicity

- White
- Irish Traveller
- Mixed/Multiple ethnic groups
- Asian/Asian British
- Indian
- Pakistani
- Bangladeshi
- Chinese
- Any other Asian background
- Black/African/Caribbean/Black British
- Arab
- Prefer not to say
- Any other ethnic group (please write in)

What is your highest level of education?

- Primary in the UK (or equivalent outside the UK)
- Secondary in the UK (or equivalent outside the UK)
- Graduate or higher level in the UK (or equivalent outside the UK)

Please select the 'Somewhat agree' response option. This is an attention check

- Strongly disagree

- Somewhat disagree
- Neither agree nor disagree
- Somewhat agree
- Strongly agree

use of AI in healthcare

Artificial Intelligence (AI) refers to computer systems that can perform tasks or make decisions which would normally be performed by humans.

There are several potential applications of AI in healthcare which could support clinicians in the diagnosis and management of diseases such as cancer, which could offer significant benefits for both patients and the wider healthcare system.

It is recognised that there may be concerns from patients and the public regarding the development and use of AI technologies in healthcare which need to be understood.

Section 1: Use of AI in Cancer Diagnosis and Management

AI is gradually being applied in many different areas of healthcare and has the potential to assist in the diagnosis and management of diseases such as cancer. Traditionally, pathologists (doctors who study disease) have identified cancer cells and biomarkers using a microscope to view thin sections of tissue mounted on glass slides.

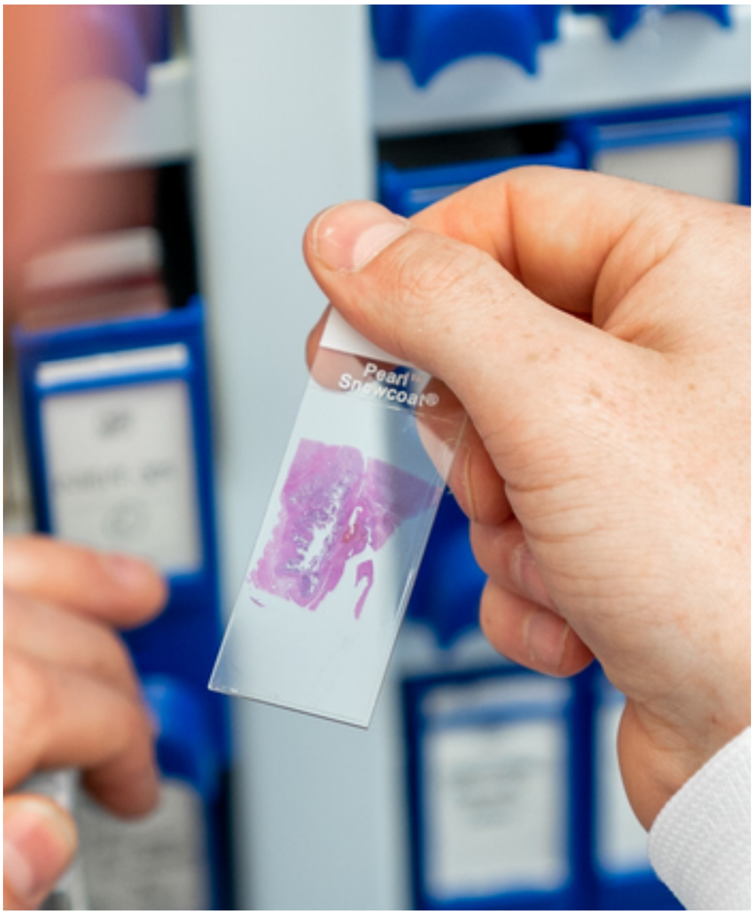

However, laboratories now use digital scanners to create a high-quality image of the glass slide which pathologists can view on a computer screen instead of a microscope.

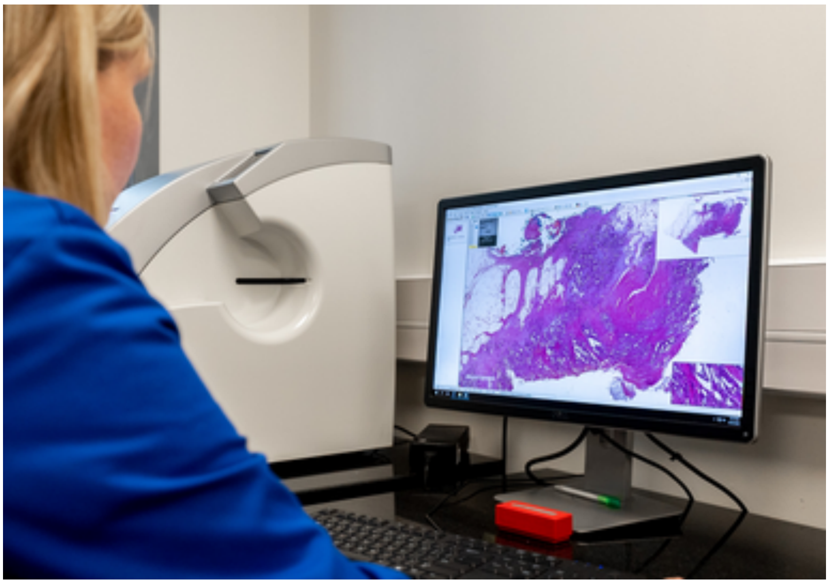

These images can be used to ‘train’ AI computer programs to assist a pathologist with their diagnosis, and in the identification of biological ‘markers’. Biological markers or 'biomarkers' are molecules in bodily fluids or tissue that can be a sign of a condition or disease and can be used to predict how well the body will respond to a treatment. AI computer programs could assist pathologists to identify biomarkers in cancer cells. This could help determine how a person’s cancer is managed. In research studies, artificial intelligence has been shown to perform equally-well, if not better, than human intelligence in this area.

In the UK, 1 in every 2 people will be diagnosed with cancer within their lifetime. We would like you to answer the following questions imagining that someone in your social circle is currently undergoing diagnosis of cancer.

Once AI technologies have been developed would you support them being used in cancer diagnosis and management?

Before taking this survey, how would you have described your awareness of Artificial Intelligence (AI) being developed for use in cancer diagnosis and management?

- Not at all aware
- Slightly aware
- Somewhat aware

- Moderately aware
- Extremely aware

Please answer the following questions on the use of AI in the **diagnosis** of cancer

I support a diagnosis of cancer being made or ruled out

	Strongly disagree	Somewhat disagree	Neither agree nor disagree	Somewhat agree	Strongly agree
by AI **only**	○	○	○	○	○
by a Pathologist **with** the assistance of AI	○	○	○	○	○
by a Pathologist **only** with **no AI** input	○	○	○	○	○

Please answer the following questions on the use of AI **in diagnosing biological 'markers' which can inform *how a person's cancer is managed.***

I support the diagnosis of biological 'markers'

Neither

	Strongly disagree	Somewhat disagree	agree nor disagree	Somewhat agree	Strongly agree
by AI **only**	○	○	○	○	○
by a Pathologist **with** the assistance of AI	○	○	○	○	○
by a Pathologist **only** with **no AI** input	○	○	○	○	○

Please rate your agreement with the following statements about the **potential impact of AI on cancer diagnosis and management**

AI will help improve cancer diagnosis and management

- ○ Strongly disagree
- ○ Somewhat disagree
- ○ Neither agree nor disagree
- ○ Somewhat agree
- ○ Strongly agree

AI will help reduce the risk of medical error or misdiagnosis

- ○ Strongly disagree
- ○ Somewhat disagree
- ○

- Neither agree nor disagree
- Somewhat agree
- Strongly agree

AI will result in less harm for patients

- Strongly disagree
- Somewhat disagree
- Neither agree nor disagree
- Somewhat agree
- Strongly agree

AI will improve efficiency and performance in the NHS

- Strongly disagree
- Somewhat disagree
- Neither agree nor disagree
- Somewhat agree
- Strongly agree

AI has the potential to improve the diagnosis and management of cancer, however patients and the public may have concerns about the development and application of AI in this context.

What do you think would help yourself or others to support the use of AI technologies in cancer diagnosis and management?
Please explain.

Section 2: Use of data in development of AI technologies

AI technologies are sometimes called ‘data driven’ technologies as they make decisions based on analysis and interpretation of vast amounts of data. To develop these technologies, researchers need access to vast amounts of data including, but not limited to, de-identified personal data from healthcare records (e.g., age, lifestyle behaviours, other health conditions).

For the NHS to realise the benefits of AI in cancer diagnosis and management, they must share this data with research and commercial organisations that are involved in developing such technologies.

This data would be de-identified to remove personal identifiers, and the sharing and use of this data would be subject to safeguards set out in the UK Data Protection Act 2018.

Research to develop these technologies requires vast amounts of data – would you support your <u>de-identified personal health data</u> being used in the development of AI

technologies to diagnose and manage cancer?

Please rate your agreement with the following statements about your **de-identified personal health data** being used in the development of AI technologies to diagnose and manage cancer.

I support my de-identified personal health data being used ***by public bodies outside the NHS*** (e.g., universities)

- Strongly disagree
- Somewhat disagree
- Neither agree nor disagree
- Somewhat agree
- Strongly agree

I trust that ***public bodies outside the NHS*** (e.g., universities) will not use my de-identified personal health data for purposes other than the development of AI technologies to diagnose and manage cancer

- Strongly disagree
- Somewhat disagree
- Neither agree nor disagree
- Somewhat agree
- Strongly agree

I support my de-identified personal health data being used ***by private***

commercial research organisations outside the NHS

- Strongly disagree
- Somewhat disagree
- Neither agree nor disagree
- Somewhat agree
- Strongly agree

I trust that ***private commercial research organisations outside the NHS*** will not use my de-identified personal health data for purposes other than the development of AI technologies to diagnose and manage cancer

- Strongly disagree
- Somewhat disagree
- Neither agree nor disagree
- Somewhat agree
- Strongly agree

I trust that the NHS have effective security and privacy protocols for sharing personal healthcare data that **will protect my anonymity.**

- Strongly disagree
- Somewhat disagree
- Neither agree nor disagree
- Somewhat agree
- Strongly agree

Section 2: Use of data in development of AI technologies

Along with de-identified personal health data, the development of AI technologies for use in cancer diagnosis and management requires data in the form of digital images of tissue slides. Traditionally, pathologists have identified cancer cells using a microscope to view thin sections of tissue mounted on glass slides.

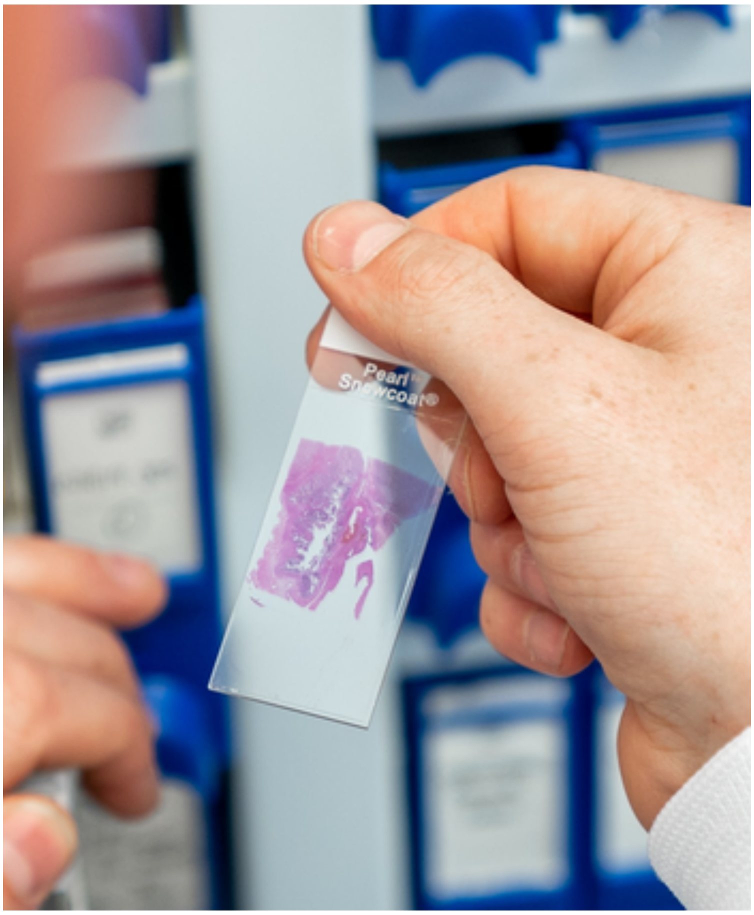

However, laboratories now use digital scanners to create a high-quality image of the glass slide which pathologists can view on a computer screen instead of a microscope. These images are a form of data that can be used to ‘train’ computer programs to assist a pathologist with their diagnosis, and in the identification of biological ‘markers’ in cancer cells that could help determine how a person’s

cancer is managed. Sharing of this data would be subject to data protection safeguards.

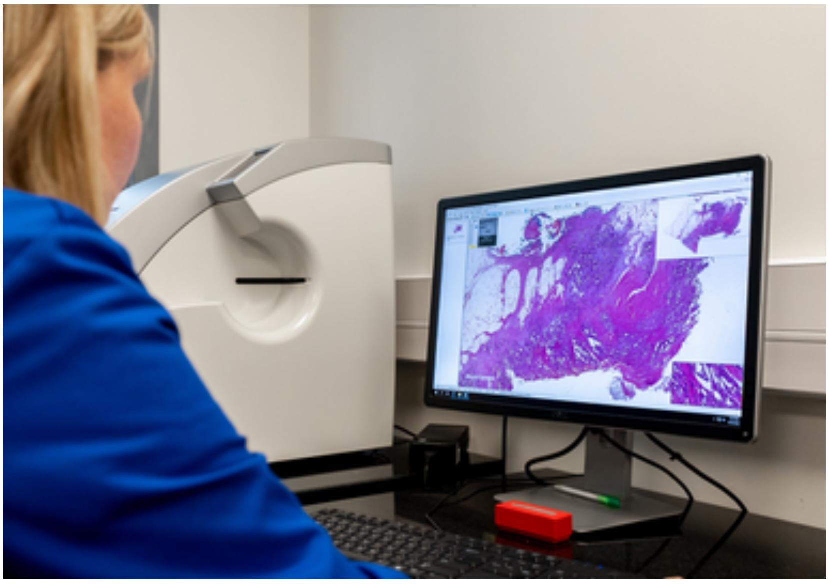

Research to develop these technologies requires vast amounts of data – would you support de-identified images of your tissue being used in the development of AI technologies to diagnose and manage cancer?

Please answer the following questions on **de-identified images of your tissue** being used in the development of AI technologies to diagnose and manage cancer.

I support de-identified images of my tissue being used ***by public***

bodies outside the NHS (e.g., universities)

- Strongly disagree
- Somewhat disagree
- Neither agree nor disagree
- Somewhat agree
- Strongly agree

I trust that ***public bodies outside the NHS*** (e.g., universities) will not use de-identified images of my tissue for purposes other than the development of AI technologies to diagnose and manage cancer

- Strongly disagree
- Somewhat disagree
- Neither agree nor disagree
- Somewhat agree
- Strongly agree

I support de-identified images of my tissue being used ***by private commercial research organisations outside the NHS***

- Strongly disagree
- Somewhat disagree
- Neither agree nor disagree
- Somewhat agree
- Strongly agree

I trust that ***private commercial research organisations outside the***

NHS will not use de-identified images of my tissue for purposes other than the development of AI technologies to diagnose and manage cancer

- ○ Strongly disagree
- ○ Somewhat disagree
- ○ Neither agree nor disagree
- ○ Somewhat agree
- ○ Strongly agree

I trust that the NHS have effective security and privacy protocols for sharing image data that **will protect my anonymity.**

- ○ Strongly disagree
- ○ Somewhat disagree
- ○ Neither agree nor disagree
- ○ Somewhat agree
- ○ Strongly agree

Section 2: Use of data in development of AI technologies

AI has the potential to improve the diagnosis and management of cancer, however patients and the public may have concerns about the development and application of AI in this context.

What do you think would help yourself or others to support the use of de-identified **images of tissue and personal health data** in the development of AI technologies to diagnose and manage cancer?

Please explain.

Which, if any, do you consider to be potential risks of using AI in cancer diagnosis and management? (select all that apply)

- ☐ Risk of harm
- ☐ Risk of error or misdiagnosis
- ☐ Lack of oversight/regulation
- ☐ Lack of accountability for decision making
- ☐ Loss of privacy
- ☐ Lack of personal interaction
- ☐ None of the above
- ☐ Other (write in)

Which, if any, do you consider to be potential benefits of using AI in cancer diagnosis and management? (select all that apply)

- ☐ More accurate decision making
- ☐ More efficiency/money savings for the NHS
- ☐ Less risk of harm
- ☐ Less risk of error or misdiagnosis
- ☐ More time freed up for staff to work on other tasks
- ☐ None of the above

☐ Other (write in) ________

End of survey message

Participant Debrief

Public awareness and attitudes toward the use of Artificial Intelligence in Cancer Diagnosis and Management

Many thanks for making a difference by participating in our survey on public awareness and attitudes towards the use of artificial intelligence in cancer diagnosis and management. The information you have provided is important to help inform communication with the public on these approaches going forward.
We will be sharing the findings of the research publicly over the coming months.
Thanks again for supporting our research.

Research team contact
Dr Claire Lewis, Honorary Lecturer at the School of Medicine, Dentistry, and Biomedical Sciences at Queen's University Belfast on claire.lewis@qub.ac.uk

Useful resources
If you found taking part in the research distressing, we would recommend that you speak with friends and family. You may also find it useful to get in touch with your GP or contact one of the helplines listed below:

Samaritans
To talk about anything that is upsetting you, you can contact Samaritans 24 hours a day, 365 days a year. You can call 116 123 (free from any phone), email jo@samaritans.org or visit some branches in person. You can also call the Welsh Language Line on 0300 123 3011 (7pm–11pm every day).

Macmillan Cancer Support
To speak to Macmillan experts, you can call the Macmillan Support Line on 0808 808 00 00 or chat to a specialist online (macmillan.org.uk)

Please click the button below to be redirected back to Prolific and register your submission

Powered by Qualtrics
